# Tassel Removal Positively Affects Biomass Production Coupled with Significantly Increasing Stem Digestibility in Switchgrass

**DOI:** 10.1371/journal.pone.0120845

**Published:** 2015-04-07

**Authors:** Chunqiao Zhao, Xifeng Fan, Xincun Hou, Yi Zhu, Yuesen Yue, Shuang Zhang, Juying Wu

**Affiliations:** 1 Beijing Research and Development Center for Grass and Environment, Beijing Academy of Agriculture and Forestry Sciences, Beijing, China; 2 College of Agriculture and Biotechnology, China Agricultural University, Beijing, China; Oklahoma State University, UNITED STATES

## Abstract

In this study, tassels of Cave-in-Rock (upland) and Alamo (lowland) were removed at or near tassel emergence to explore its effects on biomass production and quality. Tassel-removed (TR) Cave-in-Rock and Alamo both exhibited a significant (P<0.05) increase in plant heights (not including tassel length), tiller number, and aboveground biomass dry weight (10% and 12%, 30% and 13%, 13% and 18%, respectively by variety) compared to a control (CK) treatment. Notably, total sugar yields of TR Cave-in-Rock and Alamo stems increased significantly (P<0.05 or 0.01) by 19% and 19%, 21% and 14%, 52% and 18%, respectively by variety, compared to those of control switchgrass under 3 treatments by direct enzymatic hydrolysis (DEH), enzymatic hydrolysis after 1% NaOH pretreatment (EHAL) and enzymatic hydrolysis after 1% H_2_SO_4_ pretreatment (EHAC). These differences were mainly due to significantly (P<0.05 or 0.01) higher cellulose content, lower cellulose crystallinity indexes (CrI) caused by higher arabinose (Ara) substitution in xylans, and lower S/G ratio in lignin. However, the increases of nitrogen (N) and sulphur (S) concentration negatively affects the combustion quality of switchgrass aboveground biomass. This work provides information for increasing biomass production and quality in switchgrass and also facilitates the inhibition of gene dispersal of switchgrass in China.

## Introduction

Switchgrass (*Panicum virgatum* L.) shows much potential as a sustainable herbaceous energy crop from which a renewable source of transportation fuel and/or biomass generated electricity could be derived [1]. Switchgrass, a warm-season perennial C_4_ grass native to North America from 55° N latitude to central Mexico, shows promise due to its high biomass productivity, good biomass quality, and a wide range of adaptability to marginal lands with low water, nutrients or high salt and alkali [2]. Switchgrass is mainly introduced to the northern part of China, particularly in marginal lands, not only to provide a large quantity of lignocellulosic biomass materials but also to improve the ecological environment of the marginal lands [3].

Plant trade-offs among different structures or functions are a central concept of life history evolution [4]. Generally speaking, total resources are limited; increasing resources to one function may lead to a decrease in resources to other functions. Plant sexual reproduction normally requires large amounts of resources. As a result of the costs associated with the development of reproductive structures, allocation to vegetative organs could be reduced [5]. Like most perennial clonal plants, switchgrass possesses the capacity for both sexual reproduction through seeds and clonal reproduction through vegetative propagation [6]. Switchgrass shows stronger vegetative growth at the beginning stages of growth to supply sufficient nutrition for the reproductive-floral development and seeds development and ripening. Part of the mineral nutrition and other nutrient substances flow back into the root or rhizome for better wintering after the sexual reproduction finished [7]. Hence, in switchgrass there may be trade-offs among sexual reproduction, asexual reproduction and vegetative growth in carbon resources distribution and allocation. The effects of sexual reproductive organs removal or the inhibition of sexual reproduction on the vegetative biomass production or quality have been studied in many other plants. Inhibition of sexual reproduction causes a significant increase in aboveground biomass in *Arum maculatum* L. [8] and a significant increase in millable stalk and sugar yields in sweet sorghum over those of intact plants [9]. *Helianthus tuberosus* L. with inflorescence buds removed produces more and larger tubers than those with unlimited sexual reproduction [10]. Whereas, no trade-offs in carbon resources allocation between flowering and sprouting were found in *Erica australis* [11].

All the cultivation and management practices and genetic measures were explored to improve the seeds production in food crops. Different from the traditional food crops, vegetative biomass instead of the plant seeds is maximized for the production of biomass fuels. Seeds yields vary considerably ranging from 20 to 700 kg/ha in different ecotypes of switchgrass [12] under different cultivation and management practices [13]. However, based on the previous research, it is better to harvest the aboveground biomass of switchgrass later for higher biomass quality and lower transportation costs especially in marginal lands [14] which renders that a large quantity of switchgrass seeds and part of tassels fell off onto the ground. It is difficult or almost impossible to collect seeds of switchgrass for propagation and large-scale biofuels production especially in marginal lands of China. Moreover, seeds yields of switchgrass in one hectare can meet the demands of 25 to 90 ha large-scale plantation of switchgrass according to the seeds yields of switchgrass in the United States [1,15]. In addition, switchgrass, as a perennial grass, shows good wintering ability and can live up to 15 years after plantation [16]. Hence, it is preferred that the carbon resources used in formation and development of seeds were instead transferred to vegetative growth and development in switchgrass, with concurrent greater lingocellulosic biomass production.

In this study, Cave-in-Rock (upland) and Alamo (lowland) were used to explore the effects of tassel removal on vegetative growth and biomass production as well as cell wall structures and digestibility. The results may reveal the trade-offs between sexual reproduction and vegetative growth, and also propose an optimal direction for higher biomass production and quality by inhibiting sexual reproduction through genetic modification or cultivation and management measures (utilization of hormonal substances) in switchgrass.

## Results

### The vegetative growth traits of switchgrass

In this study, tassels of Cave-in-Rock and Alamo were removed to determine the changes in carbon resources allocation and cell wall structures and components in aboveground biomass. Based on a *t-*test, there were significant differences (*P*<0.05, n = 9) in switchgrass plant heights (not including tassel length), tiller number, and aboveground biomass dry weight between TR and CK treatments (Table 1). The plant heights of TR Cave-in-Rock and Alamo were significantly (*P*<0.05) higher by 12% and 12% (Table 1) than those of CK switchgrass, respectively, and the tiller number was 13% and 30% (Table 1) significantly higher (*P*<0.05) than the control value, respectively. In addition, TR switchgrass displayed increased axillary tillers initiated from the fourth or the fifth nodes in Cave-in-Rock (data not shown), however, few were found in Alamo. The stem diameter increased significantly (*P*<0.05) by 18% in TR Alamo while only a slight (*P*>0.05) increase by 7% (Table 1) exhibited in TR Cave-in-Rock compared to the control value. A slight (*P*>0.05) increase in internodes number was also observed in TR Cave-in-Rock and Alamo (8% and 3%, respectively, *P*>0.05) compared to the control value. Hence, the dry weight of TR Cave-in-Rock and Alamo aboveground biomass notably (*P*<0.05) increased by 19% and 16% (Table 1), respectively, compared to the control value.

**Table 1:** The heights, tiller number, internodes number, stem diameter and dry weight of two ecotypes of switchgrass.

Cultivars	Treatments	Heights^#^	Tiller number	Internodes number	Stem diameter	Dry weight
		(**cm**)			(**mm**)	(**g/plant**)
**Cave-in-Rock**	CK	**83.3±15.1** ^ζ^ *	**142.8±18.7** *	6.0±0.8	5.5±0.8	**474.1±88.1** *
	TR	**93.0±11.4**	**161.1±15.7**	6.5±1.4	5.9±0.5	**562.4±69.3**
		**12**%^λ^	**13**%	8%	8%	**19**%
**Alamo**	CK	**123.6±10.5** *	**93.6±13.0** *	6.1±0.7	**5.9±0.6** *	**712.8±57.6** *
	TR	**138.4±15.2**	**122.0±24.0**	6.3±1.0	**7.0±0.3**	**824.3±75.9**
		**12**%	**30**%	3%	**18**%	**16**%

CK: control, TR: tassel removal

ζ, data represents mean ± SD value (n = 9).

*, a significant difference between CK and TR by *t-*test at *P*< 0.05 and 0.01 (n = 9).

λ, percentage of the increased level: subtraction of two samples divided by CK value at pair.

#,The length of tassel was not included into the total height of switchgrass.

**Fig 1:**
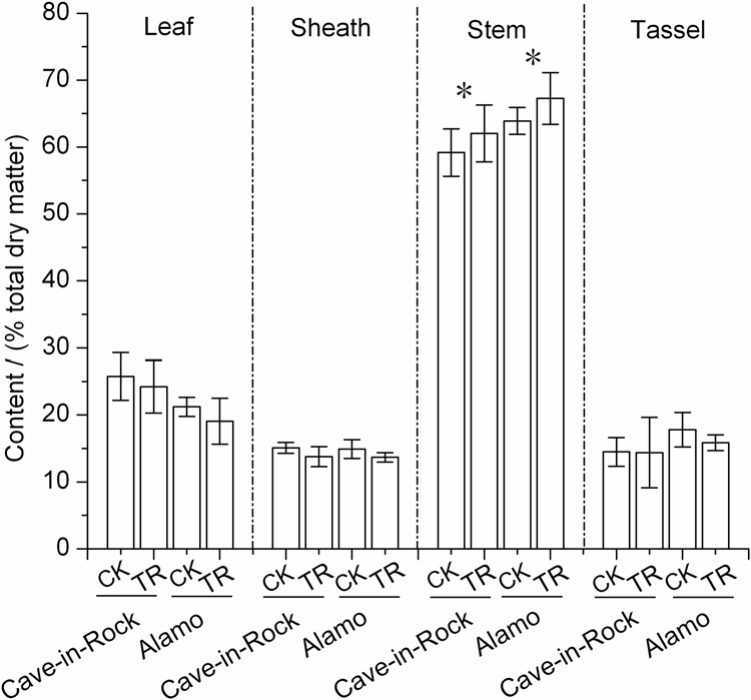
Biomass percentage of switchgrass leaves, sheaths, stems and tassels *, a significant difference in biomass percentage between CK (control) and TR (tassel removal) by *t*-test at *P*<0.05 (n = 9). The dry weight of seeds was not included into the total dry weight of switchgrass. Bar indicates SD value.

Biomass percentage of different organs in TR switchgrass exhibited some changes compared to the control value (Fig. 1). Switchgrass stems accounted for a large proportion of biomass percentage ranging from 59% to 67% (Fig. 1). Notably, the biomass percentage of switchgrass stems significantly (*P*<0.05) increased by 5% and 5% (Fig. 1) in Cave-in-Rock and Alamo, respectively, compared to the control value. However, the biomass percentage of leaves, sheaths, and tassels slightly (*P*>0.05) decreased by 6% and 10%, 9% and 8%, 1% and 11% (Fig. 1) in TR Cave-in-Rock and Alamo, respectively by variety.

### Cell wall digestibility of switchgrass stems, leaves and sheaths using 3 methods

**Fig 2:**
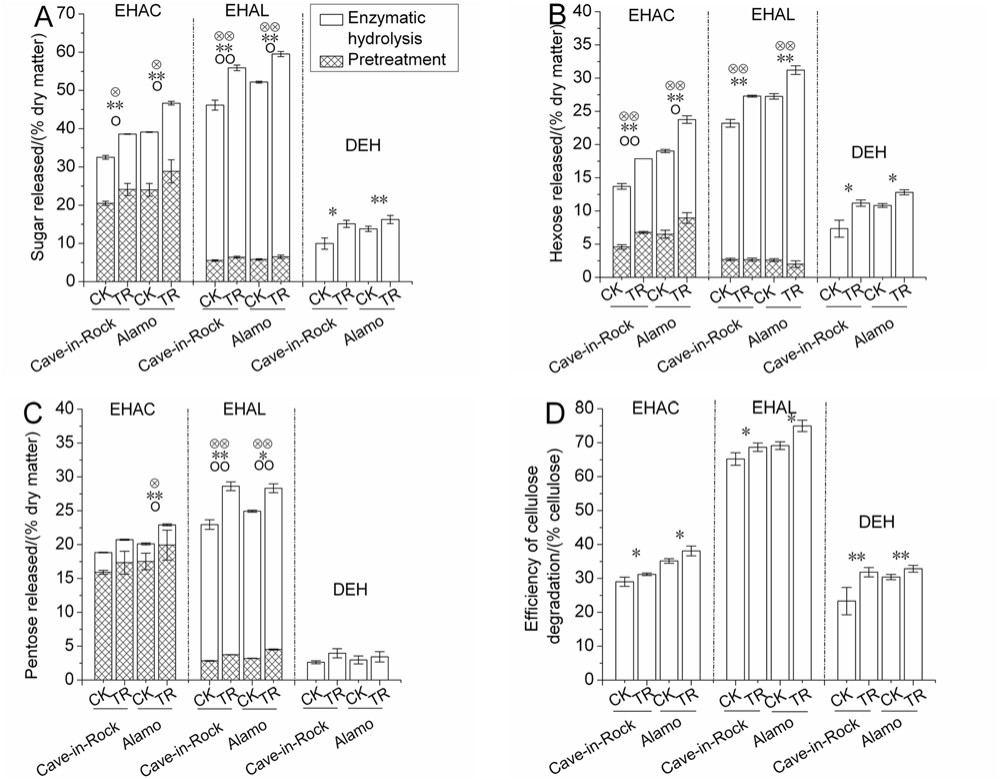
Sugar yields of switchgrass stems (A) Sugar (hexoses and pentoses) yields released from pretreatment and enzymatic hydrolysis. (B) Hexoses released from pretreatment and enzymatic hydrolysis. (C) Pentoses released from pretreatment and enzymatic hydrolysis. (D) Efficiency of cellulose degradation (% total cellulose). EHAC: enzymatic hydrolysis after 1% H_2_SO_4_ pretreatment, EHAL: enzymatic hydrolysis after 1% NaOH pretreatment. DEH: direct enzymatic hydrolysis. Efficiency of cellulose degradation = Sugar yields released from enzymatic hydrolysis / content of cellulose. ○ and ○○ indicates a significant difference in sugar (hexoses and pentoses for A, hexoses for B, pentoses for C) yields released from pretreatment between CK (control) and TR (tassel removal) by *t*-test at *P*<0.05 and 0.01 (n = 3), * and ** indicates a significant difference in sugar (hexoses and pentoses for A, hexoses for B, pentoses for C) yields released from enzymatic hydrolysis between CK (control) and TR (tassel removal) by *t*-test at *P*<0.05 and 0.01 (n = 3), ⓧand ⓧⓧ indicates a significant difference in total sugar (hexoses and pentoses for A, total hexoses for B, total pentoses for C) yields released from pretreatment and enzymatic hydrolysis between CK (control) and TR (tassel removal) by *t*-test at *P* < 0.05 and 0.01 (n = 3). Bar indicates SD value.

Different pretreatment conditions could reflect the changes in different cell wall components and structures which could better exhibit the mechanisms of changes in digestibility. In this study, 3 treatments by direct enzymatic hydrolysis (DEH), enzymatic hydrolysis after 1% NaOH pretreatment (EHAL) and enzymatic hydrolysis after 1% H_2_SO_4_ pretreatment (EHAC) were used to determine the biomass digestibility (% dry biomass weight). Based on a *t*-test between TR and CK treatments, large differences were found in stems sugar yields (Fig. 2). Total sugar yields of TR Cave-in-Rock and Alamo stems under EHAC, EHAL and DEH increased significantly (*P*<0.05 or 0.01) by 19% and 19%, 21% and 14%, 52% and 18% (Fig. 2A), respectively by variety. Under pretreatment procedure of EHAC and EHAL, sugar yields of TR Cave-in-Rock and Alamo stems were significantly (*P*<0.05 or 0.01) higher (18% and 20%, 16% and 12%, respectively by variety) than the control value (Fig. 2A). Notably, there was a significant (*P*<0.05 or 0.01) increase in sugar yields of TR Cave-in-Rock and Alamo stems by 52% and 18%, 21% and 18%, 22% and 14% (Fig. 2A) under enzymatic hydrolysis procedure of DEH, EHAC and EHAL, respectively by variety. While, few differences appeared in biomass digestibility of leaves and sheaths between TR and CK treatments (details could be found in S1 Fig.) under 3 treatments. Total sugar yields of TR Alamo leaves and sheaths increased significantly (*P*<0.05 or 0.01) by 14% and 14% (details could be found in S1 Fig.) under EHAC, respectively. Additionally, sugar yields of TR Alamo sheaths increased significantly (*P*<0.05) by 21% under pretreatment procedure of EHAC. There was also a significant (*P*<0.05) increase by 8% in sugar yields of TR switchgrass sheaths under enzymatic hydrolysis procedure of EHAL. Whereas, any other significant differences in sugar yields of switchgrass leaves and sheaths were not found (details could be found in S1 Fig.).

Large differences based on a *t*-test in hexoses yields released from pretreatment and enzymatic hydrolysis procedure of DEH, EHAC and EHAL were observed between TR and CK treatments. Hexoses yields of TR Cave-in-Rock and Alamo stems under DEH exhibited a significant (*P*<0.05) increase by 53% and 18% (Fig. 2B), respectively. With treatment by EHAC, hexoses yields of TR Cave-in-Rock and Alamo stems were significantly (*P*<0.01) higher by 48% and 38% (Fig. 2B), respectively, than the control value. Under the pretreatment procedure of EHAC, hexoses yields of TR Cave-in-Rock and Alamo stems increased significantly (*P*<0.01) by 22% and 18% (Fig. 2B), respectively. What is more, significant (*P*<0.01) increases by 22% and 18% in hexoses yields of TR Cave-in-Rock and Alamo stems, respectively, were observed under enzymatic hydrolysis procedure of EHAC. With treatment by EHAL, hexoses yields of TR Cave-in-Rock and Alamo stems exhibited significant (*P*<0.01) increases by 18% and 15%, respectively. Notably, there were significant (*P*<0.01) increases by 20% and 19%, respectively, in hexoses yields of TR Cave-in-Rock and Alamo stems under enzymatic hydrolysis procedure of EHAL. Whereas, no significant differences in hexoses yields were found under the pretreatment procedure of EHAL between TR and CK treatments. When talking about the biomass digestibility of switchgrass leaves and sheaths, there were no significant differences between TR and CK treatments under 3 treatments (details could be found in S2 Fig.).

Stems of TR Cave-in-Rock and Alamo exhibited different changes in pentoses yields compared to the control value under 3 treatments (Fig. 2C). Differences in pentoses yields were not found between TR and CK treatments under treatment of DEH. However, the pentoses yields of TR Alamo stems released from pretreatment procedure and enzymatic hydrolysis procedure of EHAC were significantly (*P*<0.05) higher by 14% and 15% (Fig. 2C), respectively, than the control value. So the total pentoses yields of TR Alamo switchgrass stems exhibited a significant (*P*<0.05) increase by 14% under EHAC. With treatment by EHAL, the pentoses yields of TR Cave-in-Rock and Alamo stems exhibited significant (*P*<0.01) increases by 32% and 24%, 41% and 10% under the pretreatment procedure and enzymatic hydrolysis procedure, respectively. Thus, there were significant increases by 25% and 14% (Fig. 2C) in total pentoses yields of TR Cave-in-Rock and Alamo stems under EHAL. While, no significant differences appeared in the pentoses yields of leaves and sheaths between TR and CK treatments, except for the sheaths of TR Alamo with a significant (*P*<0.05) increase by 9% under enzymatic hydrolysis procedure of EHAL. The total pentoses yields of TR Alamo sheaths increased significantly (*P*<0.05) by 7% under EHAL (details could be found in S3 Fig.).

In this work, efficiency of cellulose degradation (hexoses released from enzymatic hydrolysis/cellulose content) was calculated and analysed to reflect the degree of difficulty or ease in cellulose saccharification. Results showed that the efficiency of cellulose degradation of TR Cave-in-Rock and Alamo stems significantly (*P*<0.05) increased by 8% and 8%, 5% and 8%, 37% and 8% (Fig. 2D) under EHAC, EHAL and DEH, respectively by variety, compared to the control value.

Sugar yields of switchgrass stems exhibited large differences among 3 treatments. The highest hexoses yields, pentoses yields and sugar (pentoses and hexoses) yields were produced by EHAL. What is more, a very large proportion of hexoses yields, ranged from 88% to 94%, 62% to 67% (% total hexoses yields), respectively (Fig. 2B), released from enzymatic hydrolysis procedure of EHAL and EHAC. The proportion of pentoses yields released from pretreatment procedure of EHAC was 84% to 87% (% total pentoses yields) (Fig. 2C). However, things were different for pentoses yields under enzymatic hydrolysis procedure of EHAL and EHAC, with proportion ranging from 84% to 88%, 13% to 16%, respectively (Fig. 2C). Hence, the sugar (pentoses and hexoses) yields released from enzymatic hydrolysis procedure accounted for a proportion of 88% to 89%, 37% to 39% (% total sugar yields), respectively, under EHAL and EHAC (Fig. 2A). Cellulose degradation efficiency ranged from 65% to 75% (% cellulose content) under EHAL, while that ranged from 23% to 33% under DEH (Fig. 2D). The sugar yields of TR switchgrass leaves and sheaths exhibited the similar changing trend among 3 treatments (details could be found in S1–S3 Figs.).

### Cell storage polysaccharides and cell wall components of switchgrass stems

Due to the significant differences in biomass digestibility of switchgrass stems, cell wall components and cell storage polysaccharides were determined and analysed. Results showed that the cellulose content of TR Cave-in-Rock and Alamo stems significantly (*P*<0.05) increased by 12% and 9% (Table 2), respectively, compared to the control value. There were slight (*P*>0.05) decreases in the content of hemicellulose (1% and 4%) and Klason lignin (9% and 3%) in TR Cave-in-Rock and Alamo stems (Table 2). Notably, the soluble sugar content of TR Cave-in-Rock and Alamo stems increased significantly (*P*<0.01) by 179% and 85% (Table 2), respectively, and the starch content was significantly (*P*<0.01) higher by 92% and 129% (Table 2), respectively, than the control value.

**Table 2:** Three major wall polymers and storage carbohydrates of stems.

Cultivars	Treatments	Three major wall polymers/ (% dry weight)			Storage carbohydrates/ (% dry weight)	
		Cellulose	Hemicellulose	Klason lignin	Soluble sugar	Starch
**Cave-in-Rock**	CK	**31.5±1.1** ^ζ^ *	26.1±0.2	15.1±0.6	**1.8±0.3** **	**2.7±0.2** **
	TR	**35.1±0.8**	25.9±0.6	13.7±0.5	**5.0±0.4**	**5.2±0.3**
		**12%** ^λ^	-1%	-9%	**179%**	**92%**
**Alamo**	CK	**35.7±1.1** *	25.0±0.9	13.5±1.5	**2.4±0.1** **	**3.3±0.4** **
	TR	**39.0±1.1**	24.1±0.8	13.1±1.9	**4.4±0.3**	**7.5±0.5**
		**9%**	-4%	-3%	**85%**	**129%**

CK: control, TR: tassel removal

ζ, data represents mean ± SD (n = 3)

* and **, a significant difference at pair by *t*-test at *P*<0.05 and 0.01 (n = 3)

λ, percentage of the increased level at pair: subtraction of two samples divided by CK value at pair

### Cellulose characteristics, hemicellulose monosaccharide composition and lignin monomeric compositon of switchgrass stems

**Fig 3:**
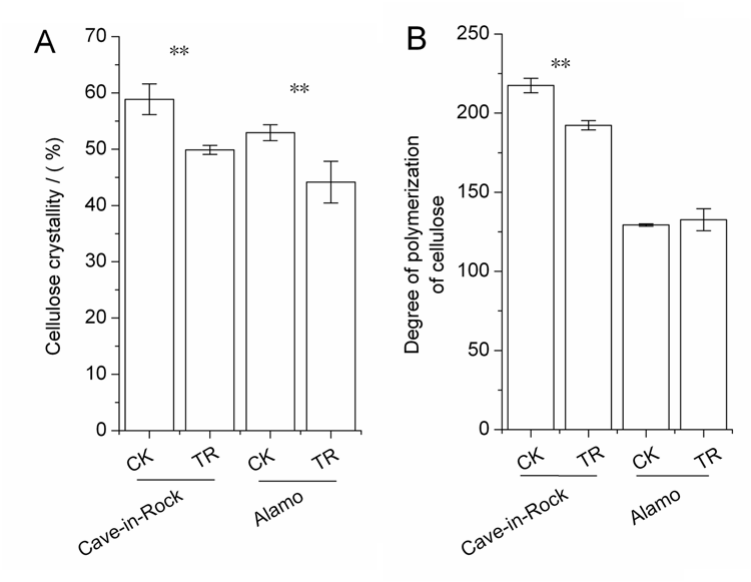
Cellulose characteristics of switchgrass stems (A) Cellulose crystallinity indexes (CrI), (B) Cellulose degree of polymerization (DP) **, a significant difference between CK (control) and TR (tassel removal) by *t*-test at *P*<0.01 (n = 3). Bar represents SD value.

There are lots of factors influencing the biomass digestibility, for example the recalcitrance of hemicellulose and lignin, cellulose characteristics in themselves, the interactions of main three components with each other. So these factors were all determined in this work. Except for the changes in cell wall components and cell storage polysaccharides, there were also some dramatic changes in fine structures of stem cell wall such as the cellulose characteristics including indexes of crystallinity (CrI) and degree of polymerization (DP), hemicellulose monosaccharide composition and lignin monomeric compositon. These fine structures were determined and analyzed in detail. Cellulose CrI of TR Cave-in-Rock and Alamo stems both decreased significantly (*P*<0.01) by 15% and 17% (Fig. 3A), respectively, compared to the control value. There was a significant (*P*<0.05) decrease by 12% (Fig. 3B) in cellulose DP of TR Cave-in-Rock stems compared to the control value, while no significant differences were found in Alamo. What is more, the monosaccharide composition of hemicellulose in TR switchgrass stems changed to some degree compared to the control value. The arabinose (Ara) content of hemicellulose in TR Cave-in-Rock and Alamo stems significantly (*P*<0.05) increased by 13% and 20% (Table 3), respectively, while xylose (Xyl) content decreased significantly (*P*<0.05) by 1% and 3% (Table 3), respectively. The ratio of Xyl/Ara (inversely reflects the Ara substitution in xylans) decreased significantly (*P*<0.05) by 13% and 19% (Table 3) in TR Cave-in-Rock and Alamo stems, respectively. Other monosaccharides of TR switchgrass hemicellulose exhibited no significant differences compared to those of CK switchgrass. Three lignin monomers *p*-Hydroxyphenyl units (H), Guaiacyl units (G), Syringyl units (S) of switchgrass stems were determined by HPLC in this work. G content of stem lignin in TR Cave-in-Rock and Alamo significantly (*P*<0.05 or 0.01) increased by 10% and 7%, respectively (Table 4). S content of stem lignin in TR Cave-in-Rock and Alamo slightly (*P*>0.05) decreased by 1% and 8% (Table 4), respectively. H content exhibited an increase by 84% (*P*<0.05) in TR Cave-in-Rock stems and 14% (*P*>0.05) in TR Alamo stems compared to the control value (Table 4). The hydroxycinnamic acids as part of lignin were also included in H content. Hence, there were decreases in S/G ratio by 11% (*P*>0.05) and 14% (*P*<0.05), increases in H/G ratio by 67% (*P*<0.05) and 7% (*P*>0.05) and increases in H/S ratio by 86% (*P*<0.05) and 24% (*P*>0.05) in TR Cave-in-Rock and Alamo stems compared to those of CK switchgrass (Table 4).

**Table 3:** Monosaccharide of hemicellulose of two switchgrass stems.

Cultivars	Treatments	Monosaccharide of hemi-cellulose /(% total monosaccharides)							Xyl/Ara
		Rha^@^	Fuc	Ara	Xyl	Man	Glu	Gal	
**Cave-in-Rock**	CK	0.3±0.03^ζ^	ND	**10.5±0.3** **	**86.0±0.3** **	0.4±0.1	1.4±0.02	1.4±0.04	**8.3±0.3** **
	TR	0.2±0.02	0.01	**11.8±0.1**	**85.0±0.1**	0.3±0.2	1.4±0.1	1.3±0.1	**7.2±0.1**
		-12%^λ^		**13%**	**-1%**	-11%	-4%	-12%	**-13%**
**Alamo**	CK	0.3±0.1	ND	**11.3±0.5** *	**85.2±0.7** *	0.3±0.1	1.5±0.02	1.5±0.03	**7.6±0.4** *
	TR	0.3±0.1	0.01	**13.6±0.3**	**82.7±0.2**	0.4±0.1	1.5±0.02	1.6±0.03	**6.1±0.1**
		8%		**20%**	**-3%**	40%	-3%	3%	**-19%**

CK: control, TR: tassel removal, ND: non-detectable

@, Rha: rhamnose, Fuc: fuctose, Ara: arabinose, Xyl: xylose, Man: mannose, Glu: glucose, Gal: galactose

ζ, data represents mean ± SD (n = 3)

* and **, a significant difference between CK and TR by *t*-test at *P*<0.05 and 0.01 (n = 3)

λ, percentage of the increased level: subtraction of two samples divided by CK value at pair

**Table 4:** Content of lignin monomers of switchgrass stems.

Cultivars	Treatments	Content of lignin monomers / (μmol /g dry matter)			S/G	H/G	H/S
		**H** ^@^	G	S			
**Cave-in-Rock**	CK	**105.3 ±18.0** ^ζ^ *	**244.9±10.8** *	159.4±5.1	0.7±0.05	**0.4±0.1** *	**0.7±0.1** *
	TR	**194.0±32.3**	**269.5±8.6**	157.3±4.2	0.6±0.01	**0.7±0.1**	**1.2±0.2**
		**84%** ^λ^	**10%**	-1%	-11%	**67%**	**86%**
**Alamo**	CK	115.0 ±11.5	**257.6±7.0** **	185.5±7.4	**0.7±0.03** *	0.5±0.04	0.6±0.04
	TR	131.1 ±16.1	**274.7±7.9**	170.5±2.3	**0.6±0.02**	0.5±0.1	0.8±0.1
		14%	**7%**	-8%	**-14%**	7%	24%

CK: control, TR: tassel removal

@, H: *p*-Hydroxyphenyl units, G: Guaiacyl units, S: Syringyl units

ζ, data represents mean ± SD value (n = 3)

* and **, a significant difference between CK and TR by *t*-test at *P*<0.05 and 0.01 (n = 3)

λ, percentage of the increased level at pair: subtraction of two samples divided by CK value at pair

### Combustion quality of switchgrass aboveground biomass

**Fig 4:**
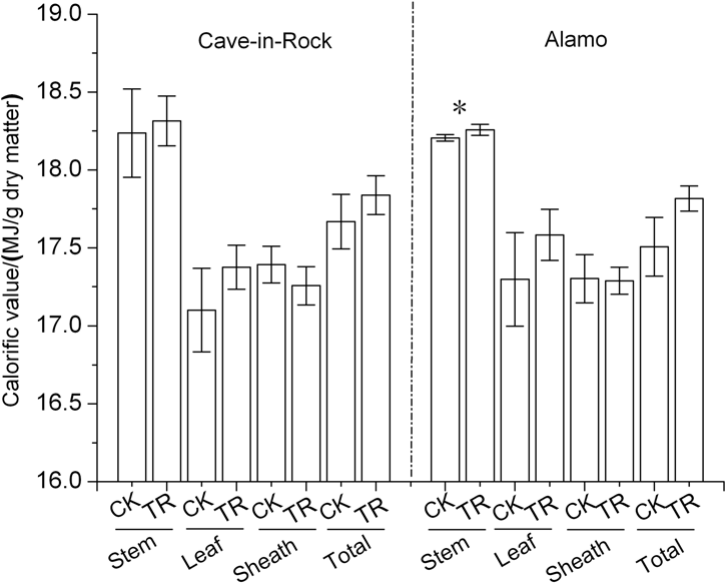
Calorific value of stems, leaves, sheaths and total aboveground biomass in switchgrass *, a significant difference between CK (control) and TR (tassel removal) by *t*-test at *P*<0.05 (n = 3). Bar represents SD value.

Combustion quality of switcgrass leaves, sheaths, stems and total aboveground biomass was determined, respectively. Switchgrass stems exhibited a higher calorific value about 18 MJ/kg (Fig. 4). There were no significant differences in calorific value of switcgrass leaves, sheaths, stems and total aboveground biomass between TR and CK treatments except for the TR Alamo stems with a significant (*P*<0.05) increase by 0.29% (Fig. 4) compared to the control value. But it is worth noting that the calorific value of different organs and total aboveground biomass almost all presented increases (*P*>0.05) trend in both ecotypes of switchgrass (Fig. 4). The calorific value increased by 1% and 2% (*P*>0.05) in aboveground biomass of TR Cave-in-Rock and Alamo compared to that of CK switchgrass. Thus, taking biomass production into consideration (Table 1), the total calorific value per plant of TR Cave-in-Rock and Alamo increased largely by 20% and 18%, respectively. The elements concentration of switchgrass aboveground biomass was also determined in this study. The nitrogen (N) and sulfur (S) concentration of TR Cave-in-Rock aboveground biomass exhibited significant (*P*<0.05 or 0.01) increases by 14% and 15% (Table 5), respectively, compared to the control value. But other elements concentration except aluminum (Al) and potassium (K) of TR Cave-in-Rock aboveground biomass exhibited slight (*P*>0.05) increases (Table 5) compared to the control value. For Alamo, a significant increase by 80% in N concentration of aboveground biomass was observed, while other elements exhibited slight (*P*>0.05) increases compared to the control value (Table 5).

**Table 5:** Elemental concentration of aboveground biomass in two ecotypes of switchgrass.

Elements	Cave-in-Rock			Alamo		
(mg /g dry matter)	CK	TR		CK	TR	
**N**	**4.7±0.3**	**5.3±0.4** ^ζ^ **	**14%** ^λ^	**6.1±0.4**	**11.0±0.9** **	**80%**
**C**	432.1±16.1	435.6±17.8	1%	436.7±4.2	437.1±6.1	
**H**	59.6±1.2	61.1±1.5	3%	59.6±0.9	60.8±1.6	2%
**S**	**0.8±0.1**	**0.9±0.1** *	**15%**	0.9±0.3	1.4±0.2*	59%
**Al**	0.1±0.01	0.1±0.01		0.2±0.02	0.2±0.01	6%
**Ca**	2.8±0.2	2.8±0.2	1%	2.7±0.3	2.8±0.5	4%
**Fe**	0.1±0.03	0.2±0.1	23%	0.1±0.03	0.1±0.02	10%
**K**	9.5±0.1	9.4±0.2	-1%	9.0±0.5	9.9±0.7	9%
**Mg**	0.9±0.1	0.9±0.1	2%	1.2±0.2	1.2±0.3	4%
**Na**	1.8±0.2	1.9±0.1	5%	2.2±0.2	2.5±0.3	12%
**P**	0.8±0.1	0.8±0.1	5%	0.7±0.1	0.8±0.1	7%
**Si**	8.2±0.6	8.7±0.7	6%	8.0±0.2	8.2±0.2	2%
**Ash**	19.6±3.5	21.6±2.9	10%	20.1±1.6	21.1±2.3	5%

CK: control, TR: tassel removal

ζ, data represents mean ± SD value (n = 3)

* and **, a significant difference at pair by *t*-test at *P*<0.05 and 0.01 (n = 3)

λ, percentage of the increased level at pair: Subtraction of two samples divided by CK value at pair

## Discussion

Switchgrass is considered as a promising bioenergy crop. However, large-scale popularization and application of switchgrass is always hindered by the biomass production and quality which are always the research targets for the increasing demands for the biomass fuels. Due to the trade-offs between sexual reproduction and vegetative growth in plants [4], removal of sexual reproductive organs may lead to increased amounts of carbon resources and nutrients available for vegetative growth [17]. Additionally, the seeds of switchgrass contribute little in the actual production process especially in marginal lands of China. However, studies on the effects of sexual reproduction on vegetative growth, particularly on the production and quality of aboveground biomass in switchgrass are very limited. Hence, this study focused on the analysis of biomass production, digestibility, cell wall components and structures, and the combustion quality in TR switchgrass.

### Effects of tassel removal on the vegetative growth of switchgrass

Trade-offs, have been defined as negative, genetically-based, associations between traits [18], reflect the resources allocation in different traits and are widely presented in plants [19,20]. As a result of tassel removal experiment, the vegetative growth of two ecotypes of switchgrass was enhanced after the sexual reproduction was inhibited by tassel removal (Table 1). More carbon resources were transferred from sexual reproduction to vegetative growth, especially to the switchgrass stems (Fig. 1). Additionally, an excess of photosynthate in TR switchgrass may stimulate the growth and development of juvenile tillers, or likely stimulate the formation of new tillers. All these growth traits lead to increases in plant tiller number and aboveground biomass dry weight (Table 1). Hence, the switch between sexual reproduction and increased vegetative plant weight shows strong trade-offs in switchgrass. On the other hand, due to the interference in growth periods by inhibition of sexual reproduction, the entire life history changed in TR switchgrass. Thus, the period of vegetative growth stages (vegetative-leaf development and elongation-stem elongation) were extended which was reflected by the increased plant heights of switchgrass and axillary tillers generated from the nodes of tillers. What is more, the apical dominance in TR switchgrass was eliminated when tassels were removed, which facilitated the vegetative growth of axillary tillers. Taken together, tassel removal positively affects the vegetative growth of switchgrass rendering higher biomass production than that of CK switchgrass, which indicated that inhibition of switchgrass sexual reproduction by minor genetic modification or hormonal substances utilization may be a promising direction for biomass production improvement in the future.

Due to the research work that the aboveground biomass of switchgrass or some other energy grasses was harvested late exhibits good quality and low costs of transportation which is more economically feasible especially in the marginal lands of China [21]. However, the dry weight of switchgrass aboveground biomass decreases in large part because of the falling and loss of leaves and tassels in late autumn or over winter [22]. So it is beneficial that the biomass percentage of switchgrass stems significantly increased (Fig. 1) after tassel removal.

### Effects of tassel removal on biomass digestibility and cell wall characteristics in switchgrass

Lignocellulosic plants especially switchgrass are increasingly being used as raw materials in the production of biomass ethanol [23]. However, lignocellulosic biomass is typically highly recalcitrant to degradation due to the highly complex structures and components of cell wall [24]. Degradation of lignocellulose with high efficiency and low cost is needed in the large-scale utilization of biomass. However, little is known about the effects of tassel removal on switchgrass biomass digestibility. Hence, the stems, leaves and sheaths of switchgrass in this experiment were subjected to 3 treatments by EHAC, EHAL and DEH, respectively. Due to the complicated structures and diverse functions of plant cell wall [25], there are several factors affecting the cell wall digestibility, such as the content of cell wall components, cellulose characteristics, lignin monomeric composition and monosaccharide composition of hemicellulose etc. Thus, the cell wall components and fine structures were analysed in detail.

TR switchgrass stems exhibits better degradability than the control switchgrass under EHAC, EHAL and DEH (Fig. 2) which indicates that tassel removal distinctly affects cell wall components or structures inducing the weakened cell wall recalcitrance. Cellulose is one of the most abundant biopolymers on earth from which a large quantity of liquid fuels could be derived. It is a high molecular weight linear polymer composed of D-glucopyranose units linked by β-1, 4-glycosidic bonds [26]. The significant increase of cellulose content (Table 3) in TR switchgrass stems contributes partly to the higher hexoses yields under enzymatic hydrolysis procedure of 3 treatments (Fig. 2B). Notably, it is quite possible that the higher content of starch in switchgrass stems caused the higher hexoses yields to some degree under pretreatment procedure of EHAC. Except for the cellulose content, it is reported that the cellulose CrI and DP serve as main factors that hinder enzymatic hydrolysis of cellulose [27]. In this work, more cellulose (higher efficiency of cellulose degradation) in TR switchgrass stems was hydrolyzed under enzymatic hydrolysis procedure of 3 treatments (Fig. 2D), to which the lower cellulose CrI of TR switchgrass stems might contribute largely in this work. Whereas, a large difference in cellulose DP was observed only in Cave-in-Rock stems which reflected the different effects of tassel removal on cellulose characteristics in different ecotypes of switchgrass. In one side, the higher cellulose degradation efficiency is induced by the changes in cellulose characteristics. On the other hand, there may be some changes in the obstacle substances (lignin or hemicellulose) which weaken the recalcitrance to enzymatic hydrolysis. Higher hexoses yields released from DEH (Fig. 2B) indicate that there are some in-situ changes in the cell wall recalcitrance structures. Acid and alkali chemicals such as H_2_SO_4_ and NaOH, with different mechanisms for biomass depolymerization [28], are extensively used in biomass pretreatments. The entire wall polymers were dissociated by breaking hydrogen and other covalent bonds under the pretreatment of alkali, whereas partial monosaccharides, oligosaccharides, and lignin monomers released by splitting strong chemical bonds under acid pretreatment [29–31]. These differences were reflected and confirmed by the different sugar (hexoses or pentoses) yields of switchgrass stems (Fig. 2) in this work. No significant differences in hexoses yields of stems exhibited between TR and CK switchgrass under pretreatment procedure of EHAL (Fig. 2B) mainly because of the similar content of hemicellulose. However, there were significant increases in hexoses yields under the enzymatic hydrolysis procedure of EHAL (Fig. 2B) which indicated that the lignin in TR switchgrass stems might be easier to be removed also confirming the results that lignin monomeric composition changed largely (Table 4).

Lignin is a complex racemic heteropolymer produced by the oxidative combinatorial coupling of main three monomers (H, G, S). Lignin content and monomers dramatically influence biomass digestibility [32]. Although the S/G ratio in lignin is recently reported to be a dual factor that affects biomass digestibility [33,34], the mechanisms remain unknown. The increases in H and G monomers content exhibited in TR switchgrass stems, while a slight decrease in S content exhibited in this work (Table 4). In other words, the lower S/G ratio might lead to the increase of stems digestibility in this study, which was consistent with research of transgenic switchgrass with higher digestibility induced by lower S/G. Whereas, the lignin content of transgenic switchgrass also decreased largely compared to the control value [35]. Lignification of plant cell walls increased drastically from elongation stage to reproductive stage. The relative S lignin content and S/G ratio increased when plants matured. It seemed that G lignin was deposited at the early stages of plant growth, and S lignin was preferentially deposited at the later developmental stages [36]. Because the vegetative growth of TR switchgrass was enhanced and extended compared to a control treatment, weaker lignification of cell wall especially the lower S/G ratio exhibited which might be an important factor responsible for the higher digestibility of TR switchgrass stems. It has been noted that the aromatic composition of lignin of monocot plants is characterized by the presence of H unit [37]. H unit only comprises a small portion of total lignin when compared with S and G lignin in most monocot species [38]. However, a relative higher H content was determined in this work which was likely due to the hydroxycinnamic acids as part of lignin. Different monomeric compositions of lignin affected the extraction of lignin-hemicellulose complexes due to different covalent bonds of ether linkages and ester linkages, which leads to different biomass digestibility. According to current work, G-rich lignin is more likely to be extracted by mild alkali-pretreatment for high lignocellulose digestibility in *Miscanthus* [39]. Additionally, the minor wall-networks between monolignols and interlinked-phenolics predominantly affect biomass enzymatic digestibility in *Miscanthus* [32], so it seems very important to analyse the interlinking of lignin monomers with wall polymers such as hemicellulose and cellulose. In this work, samples with lower S/G ratio exhibited lower cellulose CrI and higher digestibility which was also found in transgenic switchgrass [35]. It suggested that the S/G ratio or especially the G monomers might be related with cellulose polymers in this work. Whereas, whether there are negative effects of lignin monomers especially the G monomers on cellulose CrI and DP still remains unclear.

Hemicellulose is polysaccharides with various sugar units in plant cell walls that has β-(1→4)-linked backbones with an equatorial configuration [40]. In the secondary cell wall of *Gramineae* plants, xylans as major hemicellulose are most commonly substituted by α-L—arabinofuranosyl units on the C_2_- and / or C_3_-position in arabinoxylan, and α-D-glucopyranosyl uronic units or its 4-O-methylderivative side chains on the C_2_-position in glucuronoarabinoxylan (GAX) [41]. Hemicellulose plays an important role in cross-linked interactions with cellulose and lignin. Because cellulose tends to contain both well-ordered crystalline regions and disordered, more amorphous regions [42], the degree of Ara substitution in xylans has been considered as the key factor that positively affects biomass saccharification for Ara substitution in xylans is partially associated with cellulose in amorphous regions, which negatively affects cellulose crystallinity [43]. As described above, the cellulose crystallinity of TR switchgrass stems decreases (Fig. 3A) significantly, which indicates that the hemicellulose structures (mainly reflected by the degree of Ara substitution in this work) dramatically affects the crystallinity of cellulose, leading to higher biomass digestibility. While the relationships between hemicellulose and cellulose DP seemed ambiguous in this work.

### Effect of tassel removal on the combustion quality of switchgrass aboveground biomass

Generally, production of bioenergy crops seeks to maximize the concentration of lignocellulose in the feedstock, minimize nitrogen (N) and mineral concentration, and limit water concentration [44]. The concentration of elements usually decreases in forages as they mature because of the nutrients retranslocation [45,46]. However, TR switchgrass has a relative high concentration of elements including aluminum (Al), calcium (Ca), ferrum (Fe), magnesium (Mg), sodium (Na), phosphorus (P) and silicon (Si) with sulphur (S) and N the most obvious. The increased concentration of alkali metals in the switchgrass biomass negatively affects biofuel quality because these can increase the formation of fusible ash, causing slagging and fouling of boilers used in direct combustion [47]. The significant increase in N concentration may render that more and more N fertilizer is extracted out of the soil and required for the switchgrass growth inducing an increase in the costs of biomass production. In addition, the significant increases in S and N concentration render more oxysulfide and oxynitride gas released during combustion which adversely affects the environment during combustion utilization. From another perspective, slight higher elements concentration may reflect that the nutrients retranslocation were influenced to some degree by the continuous vegetative growth caused by the later-finished life cycle in TR switchgrass. As a perennial grass, growing ages are related with rhizome and root which owns good ability of wintering in switchgrass. But it is still unclear that whether the slight higher elements concentration could negatively affect the growth limit of switchgrass in the long term.

### Inhibition of sexual reproduction for cost effective biomass materials production

Switchgrass shows huge potential as an energy grass. Biomass production and quality is very important, especially for viable sustainable development of bioenergy in China. Cultivation and management practices can improve the biomass production while huge amounts of investments are needed especially in the marginal lands of China. What is more, it is difficult to genetically modify the plant cell walls to improve biomass digestibility without any negative influences on growth and development of switchgrass. Additionally, it still needs further investigation on how to improve the switchgrass biomass production and quality simultaneously in marginal lands. In this study, tassel removal positively affects the biomass production and digestibility in two ecotypes of switchgrass. The carbon resources were diverted from sexual reproduction to vegetative growth and the cell wall components especially the structures changed largely. It seems to be a promising direction to improve the biomass production and quality by inhibiting of sexual reproduction in switchgrass. Hence, this work proposes a new direction for higher biomass production and quality by minor genetic modification of sexual reproduction, or hormonal regulation which can inhibit the sexual reproduction in switchgrass. Furthermore, this work also facilitates the inhibition of gene dispersal of switchgrass in China.

## Conclusions

Tassel removal of two ecotypes of switchgrass distinctly changes biomass production and biomass quality of switchgrass. TR switchgrass has increased aboveground biomass production compared to the control value. Furthermore, the stems of TR switchgrass exhibit more favorable biomass digestibility due to higher cellulose content, lower lignin monomer S/G ratio, and notably a lower cellulose crystalinity indexes caused by higher Ara substitution in xylans compared to those of CK switchgrass. However, the increases of N and S concentration negatively affect the combustion quality of TR switchgrass aboveground biomass. Hence, minor genetic modification on switchgrass sexual reproduction or on the degree of Ara substitution in cell wall may significantly improve aboveground biomass production of switchgrass and enhance biomass digestibility in order to optimally facilitate ethanol production. Furthermore, this work also facilitates the inhibition of gene dispersal of switchgrass in China.

## Materials and Methods

### Plant materials and experiment design

The tassel removal experiment was conducted in the energy grass base of Beijing Research & Development Center for Grass and Environment, Beijing Academy of Agriculture and Forestry Sciences (39° 34’ N, 116° 28’ E). The experimental field, with consistent terrain, is located in the warm temperate continental monsoon climate with an average elevation of 50 m, an annual average temperature of 12–17°C, 400–600 mm annual rainfall, a frost-free period of 90–200 d and ≥ 10°C accumulated temperature of 4 200°C. The soil of experimental field was characterized by a uniform pH of 7.6 and fertility, with 15.2 g/kg of organic matter, 84.0 mg/kg of available N, 129.0 mg/kg of available K, and 16.5 mg/kg of available P.

Cave-in-Rock (Octoploid) and Alamo (Tetraploid) were used in this article. The seeds of the two switchgrass were bought from America 2003 and planted in Beijing till now. The seeds used in this experiment were harvested in 2011 manually. In late April 2012, nearly 100 switchgrass individuals with consistent nutrient soil clods, each consisting of a single tiller of 25 ± 0.6 cm in heights (mean ± s. e., n = 20), of each ecotype of switchgrass were taken outside from greenhouse and planted in a 1 m plant spacing and 1 m row spacing in 6 blocks. And the two ecotypes of switchgrass were planted in 2012 for yield trials and variety comparative test without any treatments in 2012, and then were treated in 2013 for this experiment. The cultivation and management practices were consistent among all individuals of switchgrass. Switchgrass individuals with consistent growth status were chosen as the experiment materials. In about mid-June 2013, the switchgrass tassels of each ecotype were pulled out at or near tassel emergence manually randomly selecting 3 blocks with the other 3 blocks remaining intact as controls. The tassels emerged again on the new tillers and auxiliary tillers which were pulled out till no tassels appeared. During early November 2013, after the first killing frost, aboveground biomass of CK and TR switchgrass individuals in 3 blocks was harvested, respectively, taking 9 individuals from one block as one replicate. When harvested, the leaves of Alamo were killed by frost obviously different from Cave-in-Rock with natural withering. The stubble heights of switchgrass were ~5 cm (with no more than 5 tillers 5–10 cm), maintaining the similar heights among switchgrass individuals. The samples were washed clean with tap water and then fixed at 105°C for 20 min. Then the leaves, sheaths and stems were separated and dried at 50°C until constant weight. After that, the leaves, sheaths, and stems of 9 individuals in one replicate were mixed, respectively, and then crushed using a micromill (purchased from Tianjin TEST instrument co., LTD, Tianjin, China) for 3 min and sieved using a sifter (40 meshes, 0.45 μm). Samples remaining in the sifter were crushed further with a Traditional Chinese Medicine (TCM) crusher manually until all of the samples went through the sifter. The compositional analysis, biomass digestibility and elemental analysis were conducted in triplicate taking samples powder of different organs from 9 switchgrass individuals in one block as one replicate.

### Plant growth traits measurements

Plant heights were determined from the soil surface to the bottom of tassels with 9 replicates. Diameter of the middle of the third internodes was measured using an electronic vernier caliper (purchased from Mahr Co., Ltd., Suzhou branch, China) to obtain the stem diameter with 9 replicates. The basal stems were counted manually to acquire the tiller number with 9 replicates and five tillers of each treatment of switchgrass were taken out randomly for the internodes number. Dry weight of total aboveground biomass and different organs were weighed using analytical balance (SHIMADZU BL-3200H) with 9 replicates before the samples were mixed and crushed. Seeds of switchgrass were harvested and the glumes and husks were removed manually.

### Analysis of biomass digestibility

Ten milliliters of 1% (v / v) H_2_SO_4_ or 1% (w / v) NaOH was added to a 0.5 g sample in a 15- mL centrifuge tube for the acid or alkali pretreatment, respectively. For the acid pretreatment, the tubes well shaken were heated at 121°C for 20 min in an autoclave (15 psi) and then were shaken in a table concentrator at 150 rpm and 50°C for 2 h. For the alkali pretreatment, the tubes were only shaken in a table concentrator at 150 rpm and 50°C for 2 h. Sample with 10 mL of distilled water served as a control. Afterwards, the tubes were cooled with tap water and centrifuged at 4 000 rpm for 10 min. The supernatant was obtained and placed in -20°C for the determination of sugar yields. Residues were washed 6 times with 10 mL of distilled water and 2 times with 10 mL of cellulase buffer (0.2 M acetic acid-sodium acetate, pH 4.8) for enzymatic hydrolysis.

Then 10 mL of 0.2% (w/ v) mixed cellulase buffer solution (containing ≥ 6.5×10^4^ U of β-glucanase, ≥ 700 U of cellulase, and ≥ 10 × 10^4^ U of xylanase from Imperial Jade Biotechnology Co., Ltd., Yinchuan, China) was added to the residues in the centrifuge tubes. The tubes were shaken in a table concentrator at 150 rpm and 50°C for 48 h. The tubes were then briefly placed in boiling water to deactivate the cellulases, and cooled with tap water, and then centrifuged at 4 000 rpm for 10 min. The supernatant was combined and placed at -20°C for the determination of sugar yields. Tubes with 10 mL distilled water served as controls. The sugar yields were calculated as the following formulas:

$$\text{Pentoses yields}={\text{S}}_{\text{c5}}\text{ / DW×100\%}$$

$$\text{Hexoses yields}={\text{S}}_{\text{c6}}\text{ / DW×100\%}$$

$$\text{Sugar yields}={\text{(S}}_{\text{c5}}+{\text{S}}_{\text{c6}}\text{) / DW×100\%}$$

Where the S_C5_ represents the pentoses released from pretreatment or enzymatic hydrolysis procedure. S_C6_ represents the hexoses released from pretreatment or enzymatic hydrolysis procedure. DW represents the dry weight of samples used for determination.

### Colorimetric assay of hexoses and pentoses

A UV—vis opectrophotometer (756 PC, Shanghai Hongji Instruments Co., Ltd., Shanghai, China) was used for absorbance reading values. The anthrone/H_2_SO_4_ method [48] was used to determine hexoses and the orcinol/HCl method [49] was used to determine pentoses. The details of the determination method were carried out according to Xu et al [50]. Anthrone and orcinol were purchased from Sigma-Aldrich Co., LLC and ferric chloride was obtained from Sinopharm Chemical Reagent Co., Ltd. D-glucose and D-xylose purchased from Sigma-Aldrich Co., LLC were used as standards for creating standard curves, with *R* ^*2*^ higher than 0.9990, of hexoses and pentoses.

### Carbohydrate polymers and plant cell wall fractionation and determination

Method as described by Peng et al. [51] and Xu et al. [50] was used to fractionate the cell wall of switchgrass stems with minor modification. In detail, about 0.1 g sample powder was added to porcelain mortar with 2 mL potassium phosphate buffer (0.2 mol /L, pH 7.0) in it and the samples were grinded till homogenate like. Then the turbidity solution was transferred to 15 mL centrifuge tubes with 10 mL turbidity solution, and was centrifuged at 4 000 rpm for 5 min. The supernates were combined and determined, as described above, as the content of total soluble sugar. Then 10 mL chloroform-methanol (1:1, v/v) was added into the tubes and the tubes were shaken up at 150 rpm, 50°C in a shaker for 1 h to remove the lipids. After that, 10 mL superdimethylsulphoxide (DMSO) water (9:1, v/v) was added. Likewise, the supernates were combined as total starch. Before the determination of starch, the solution was dialyzed using dialysis bags (3 cm × 20 cm, molecular weight cut-off 3500) for 24 h. The distilled water was changed every 3 h during the dialysis period. Then the white flocculus from the extracted solution was collected and redissolved using 10 mL 67% H_2_SO_4_. The content of hexoses was determined using method as described above as total content of starch. Afterwards, 10 mL 0.5% (w/v) ammonium oxalate was added to extract pectin. And 10 mL 4 M KOH with 1 mg/mL sodium borohydride was added and the solution was neutralized, dialyzed as described above and determined as the total content of hemicellulose. The last step, H_2_SO_4_ (67%, v/v) was added to hydrolyse the cellulose in the residues. Total lignin was determined using a two-step acid hydrolysis method in accordance with the Laboratory Analytical Procedure of the National Renewable Energy Laboratory [52].

### Monosaccharide assay of stem hemicellulose by GC/MS

Method described in Xu et al. [50] was used for determination of hemicellulose monosaccharides. In short, supernatants from the 4 M KOH fraction were dialyzed using the dialysis bags as described above. The samples from the dialyzed KOH-extractable supernatant and the non-KOH-extractable residues were hydrolyzed for free monosaccharides release. After that, a GC/MS instrument (Agilent 5977 A GC/MSD) was used to determine the contents of monosaccharides using Myo-inositol as the internal standard material which was purchased from Sigma-Aldrich Co. LLC.

### Lignin monomers determination of stems by HPLC

The alkaline nitrobenzene oxidation (NBO) method was used to analyze the monolignin proportion as described by Wu et al. [53]. HPLC (Waters e2695 Alliance) with a column of Kromat Universil C_18_ (4.6 mm × 250 mm, 5 μm) was used to analyze the lignin monomers using *p*-Hydroxybenzaldehyde (H), Vanillin (G) and Syringaldehyde (S) (purchased from Sinopharm Chemical Reagent Co., Ltd.) as standard chemicals and ethyl vanillin as the internal standard material. The column was operated at 30°C with a CH_3_OH : H_2_O : CH_3_COOH (23 : 76 : 2, v / v / v) carrier liquid at a flow rate of 1.0 mL/min. The calibration curves of all analytes routinely yielded correlation coefficients 0.999 or higher.

### Determination of stems cellulose characteristics

An XRD instrument (SHIMAZDUO XRD-6100) was used to detect cellulose CrI of raw materials as described by Zhang et al. [54]. The degree of polymerization of raw materials cellulose was determined using the copper ethylenediamine method [55]. An Ubbelohde Viscometer with a 0.88 mm inner capillary diameter was purchased from Beijing glass instrument factory. Copper ethylenediamine (analytical reagent) was provided by China National Pulp and Paper Research Institute. The intrinsic viscosity was calculated by interpolation using the USP table (USP35, 2012) that lists the predetermined values of the product of intrinsic viscosity and concentration.

### Calorific value determination of aboveground biomass

A calorific value analysis instrument (purchased from IKA, C2000, Staufen, Germany) was used to determine the calorific value of samples. 0.5 g sample powder pressed into tablet was put in burning cup linked to a wire electrode using cotton thread with a known heat value. Then the oxygen bomb containing 10 mL of distilled water was gently screwed tight and was hung on the main engine. Oxygen filling occurred automatically with pressure of 3 MPa of oxygen. Benzoic flakes provided by IKA were used as standard materials to calibrate the instrument and make standard curves with correlation coefficients higher than 0.9990.

### Ash and elements analysis of aboveground biomass

The ash content was determined using a box-type Muffle furnace (SX_2_-4-10, Tianjin Zhonghuan Experimental Furnace Co., Ltd., Tianjin, China). About 5 g sample in a porcelain crucible was preheated on an electric heat plate in a ventilated kitchen until smoke was no longer emitted and then was transferred into the Muffle furnace for 3 h at 550°C. Then the porcelain crucible was taken out and weighed after cooling to room temperature.

Total C, H, N, and S contents were analyzed using a Vario Macro CHNS instrument (purchased from Elementar Analysensysteme GmbH, Hanau, Germany). In detail, about 50 mg of sample powder was packaged closely using a silver paper and then was compacted tightly, forming a tablet without any sample powder or impurities outside. Afterwards, they were put into the sample plate for the determination by the instrument automatically. The temperature of the combustion tube was preheated to ~1 150°C, and the temperature of the reduction tube was controlled at ~850°C. The pressure of the helium and oxygen was controlled at ~1 200–1 250 mba and 0.2–0.22MPa. Phenylalanine as the standard material was used to make standard curves of C, H, N, and S in applicable concentration ranges for plant materials and routinely yielded correlation coefficients of 0.999 or higher. About 50 mg phenylalanine was used every 20 samples to make sure the correlation coefficient varied within 0.9–1.1. An inductively coupled plasma instrument (ICP) was used to analyze the switchgrass mineral elements [56].

### Statistical analysis

Data was subjected to the analysis of *t*-test using Excel 2007. The significance was tested at *P*<0.05 and 0.01 levels. Standard errors were provided in all tables and figures as appropriate. The figures were drawn using Origin 8.5.

## Supporting Information

S1 Fig Sugar yields released from pretreatment and enzymatic hydrolysis of switchgrass leaves and sheaths. EHAC: enzymatic hydrolysis after 1% H_2_SO_4_ pretreatment, EHAL: enzymatic hydrolysis after 1% NaOH pretreatment. DEH: direct enzymatic hydrolysis. ○, a significant difference in sugar yields released from pretreatment between CK (control) and TR (tassel removal) by *t*-test at *P*<0.05 (n = 3), *, a significant difference in sugar yields released from enzymatic hydrolysis between CK and TR by *t*-test at *P*<0.05 (n = 3), ⓧ and ⓧⓧ, a significant difference in total sugar yields released from enzymatic hydrolysis and pretreatment between CK and TR by *t*-test at *P*<0.05 and 0.01 (n = 3). Bar indicates SD value.
(TIF)

S2 Fig Hexoses yields released from pretreatment and enzymatic hydrolysis of switchgrass leaves and sheaths. EHAC: enzymatic hydrolysis after 1% H_2_SO_4_ pretreatment, EHAL: enzymatic hydrolysis after 1% NaOH pretreatment. DEH: direct enzymatic hydrolysis. Bar indicates SD value (n = 3).
(TIF)

S3 Fig Pentoses yields released from pretreatment and enzymatic hydrolysis of switchgrass leaves and sheaths. EHAC: enzymatic hydrolysis after 1% H_2_SO_4_ pretreatment, EHAL: enzymatic hydrolysis after 1% NaOH pretreatment. DEH: direct enzymatic hydrolysis. **, a significant difference in pentoses yields released from enzymatic hydrolysis between CK and TR by *t*-test at *P*<0.01 (n = 3), ⓧ, a significant difference in total pentoses yields released from pretreatment and enzymatic hydrolysis between CK and TR by *t*-test at *P*<0.05 and 0.01 (n = 3). Bar indicates SD value.
(TIF)
